# Patient-centered care for patients requiring dialysis during disasters and public health emergencies: a scoping review

**DOI:** 10.1186/s12245-026-01253-7

**Published:** 2026-05-29

**Authors:** Amir Khorram-Manesh, Eric Carlström

**Affiliations:** 1 Institute of Clinical Sciences, Sahlgrenska Academy, University of Gothenburg, Gothenburg, Sweden; 2 Center for Disaster Medicine, University of Gothenburg, Gothenburg, Sweden; 3 Gothenburg Emergency Medicine Research Group, Sahlgrenska University Hospital, Gothenburg, Sweden; 4 Institute of Health and Care Sciences, University of Gothenburg, Gothenburg, Sweden

**Keywords:** Patient-centered care, Dialysis, Disaster, Emergency preparedness, Public health emergency, Vulnerable populations, Scoping review

## Abstract

**Background:**

Disasters and public health emergencies disrupt health systems, threaten continuity of care, and disproportionately affect medically vulnerable populations. Patients requiring dialysis are especially at risk because their survival depends on regular access to complex, resource-intensive treatment. While disaster nephrology literature increasingly addresses preparedness and resilience, it remains unclear how explicitly this literature addresses patient-centered care.

**Objective:**

To map the literature on patient-centered care for patients requiring dialysis during disasters and public health emergencies, with attention to preparedness, continuity of care, communication, access, and vulnerable populations.

**Methods:**

A scoping review was conducted using searches in Web of Science, Scopus, and PubMed. Search terms combined concepts related to disaster, emergency, patient-centered care, vulnerability, dialysis, and alternative care facilities. Records were screened for relevance to dialysis care in disasters or public health emergencies, or for conceptual relevance to patient-centered dialysis care in disrupted settings. Both authors conducted screening, eligibility assessment, and data charting, using a predefined set of inclusion criteria and a consistent conceptual framework. The search was intentionally focused to prioritize literature most directly relevant to dialysis-dependent patients in disaster and public health emergency contexts. Forty-three studies were included.

**Results:**

The included studies comprised reviews, scoping reviews, observational studies, qualitative studies, case studies, program reports, pilot studies, trials, and conceptual papers. The literature clustered around six themes: preparedness for dialysis patients and providers; continuity of care and treatment access; infrastructure, logistics, and resilience; patient awareness, communication, and lived experience; public health emergency adaptations, particularly during COVID-19; and broader frameworks for disaster risk reduction in kidney care. Although relatively few studies explicitly used the term patient-centered care, many addressed closely related domains, including communication, care planning, family involvement, psychosocial support, access, and continuity. Taken together, the findings suggest that patient-centered dialysis care in emergencies is shaped by broader health system factors, including preparedness, coordination, service design, and equity.

**Conclusions:**

Patients requiring dialysis are among the most vulnerable groups during disasters and public health emergencies. Existing literature provides a strong foundation in preparedness and continuity of treatment but addresses patient-centered care more often implicitly than explicitly. Future research should develop and test patient-centered indicators for dialysis care in emergencies, including communication, shared planning, caregiver involvement, psychosocial support, and the role of alternative care facilities.

**Supplementary Information:**

The online version contains supplementary material available at 10.1186/s12245-026-01253-7.

## Introduction

Disasters and public health emergencies (DPHEs) place extraordinary pressure on health systems and can rapidly undermine the continuity, accessibility, and quality of care. Damage to physical infrastructure, interruption of transport routes, shortages in medical supplies, loss of electrical power and water, and workforce depletion can all compromise the delivery of both routine and specialized health services [1]. Such disruptions may arise in a wide range of events, including hurricanes, floods, earthquakes, wildfires, pandemics, infectious disease outbreaks, and other large-scale emergencies. In these contexts, health systems often shift toward crisis-oriented priorities such as surge capacity, triage, stabilization, and the maintenance of essential services [2]. While these priorities are necessary, they can also narrow the focus of care toward operational survival alone. As a result, DPHEs reveal an important tension within emergency response: the need not only to preserve health system function, but also to maintain care that remains responsive to patients’ needs, preferences, dignity, and continuity [3].

The impact of these disruptions is not evenly distributed across populations. Vulnerable groups are consistently recognized as being at increased risk of harm during DPHEs because pre-existing health and social disadvantages are often magnified under crisis conditions [1, 4]. Importantly, vulnerability should not be understood only in terms of disease burden or biological frailty. It is also shaped by dependence on ongoing treatment, reduced mobility, unstable housing, limited financial or social resources, caregiver availability, communication barriers, and reliance on complex care systems [5–7]. In other words, vulnerability is both medical and structural. Patients whose lives depend on regular, technology-supported, and system-dependent treatment become especially exposed when emergency conditions disrupt the networks that sustain their care. This makes the identification and protection of vulnerable groups a central ethical and practical concern in disaster medicine.

Patients requiring dialysis represent one of the clearest examples of this compounded vulnerability. Both maintenance hemodialysis and peritoneal dialysis depend on a tightly coordinated chain of resources and services, including trained professionals, functioning treatment sites, specialized equipment, clean water, reliable electricity, consumables, transport systems, and ongoing communication between providers and patients [8, 9]. When even one part of this chain fails, dialysis care may be delayed, interrupted, or delivered under suboptimal conditions. For patients with end-stage kidney disease, such interruptions are not merely inconvenient; they may quickly become life-threatening [8]. Documented consequences include delayed or missed treatments, emergency dialysis starts, fluid overload, electrolyte imbalance, hospitalization, and death [10–13]. The disaster nephrology literature has repeatedly shown that major emergency events such as Hurricane Katrina, Hurricane Sandy, and Hurricane Maria had profound effects on dialysis continuity, patient outcomes, and health system capacity [10, 12, 14–17]. These experiences underscore that dialysis-dependent populations are not simply one vulnerable group among many, but a population whose survival is uniquely tied to uninterrupted and coordinated care delivery.

At the same time, continuity of treatment, while essential, does not by itself capture the full scope of what high-quality dialysis care should mean during DPHEs. A narrow focus on whether dialysis was technically delivered may overlook equally important patient-centered dimensions of care. Patient-centered care involves understandable and timely communication, respect for patient preferences and values, shared planning where possible, psychosocial support, family and caregiver involvement, and adaptation of services to patients´ lived realities [3]. In the emergency dialysis context, these dimensions become especially salient. For example, patients may need clear explanations of altered treatment schedules, guidance on evacuation or transport, support in navigating unfamiliar care sites, and reassurance that their concerns and preferences remain recognized despite crisis conditions. Questions also arise regarding whether emergency plans are understandable, whether alternative treatment arrangements are practically accessible, whether caregivers or support persons are included, and whether the burden of uncertainty, displacement, and treatment disruption is acknowledged in care planning [18–22]. Thus, patient-centered care is not secondary to emergency dialysis care; rather, it is a crucial lens through which the adequacy and humanity of that care should be evaluated.

This issue is particularly important because a growing body of literature has addressed emergency preparedness for dialysis patients and kidney care systems, but often from an operational or clinical preparedness perspective rather than an explicitly patient-centered one [23, 24]. Existing work has examined disaster planning for patients with kidney disease, emergency recommendations from professional and public health bodies, and preparedness strategies for both patients and providers [25–28]. Other studies have emphasized resilience in kidney care systems and broader approaches to disaster risk reduction and management [7, 29]. In parallel, the COVID-19 pandemic has expanded the literature by highlighting issues such as patient experience, quality of life, telehealth, infection control, access disruption, and adaptation by both patients and professionals [22, 30–32]. Collectively, these studies provide an important foundation for understanding dialysis care in disrupted environments. However, the literature remains dispersed across nephrology, nursing, disaster medicine, public health, and health services research, and it is still unclear how consistently patient-centered care is defined, recognized, or operationalized in this body of work.

Taken together, these gaps highlight the need for a clearer synthesis of how patient-centered care for dialysis-dependent patients is addressed in DPHEs. Beyond continuity of treatment and logistical preparedness, it is important to understand whether the literature also addresses communication, patient preferences, caregiver involvement, psychosocial needs, and equitable access under disrupted conditions. Viewed through a person-centered health systems lens, emergency dialysis care is not only a matter of clinical continuity, but also of how service design, coordination, and system capacity support or constrain care for a highly vulnerable population. This perspective is also relevant to broader health system goals, including Sustainable Development Goal (SDG) 3 on good health and well-being and SDG 10 on reduced inequalities, as disruptions in dialysis care are likely to disproportionately affect patients with fewer social, financial, transport, or caregiving resources [33]. For the purposes of this review, patient-centered care was understood as encompassing communication, continuity of care, patient preferences, caregiver and family involvement, psychosocial support, equitable access, and adaptation of services to patients´ lived circumstances [3].

Accordingly, this review examines how the literature conceptualizes and addresses patient-centered care for patients requiring dialysis during DPHEs, with a focus on preparedness, continuity of care, access, communication, and the needs of vulnerable patients.

## Materials and methods

### Study design

This study used a scoping review design to map the extent, range, and nature of the literature relevant to patient-centered care for patients requiring dialysis during DPHEs. No protocol was prospectively registered for this review; however, the review was conducted according to scoping review methodology and reported with reference to PRISMA-ScR guidance. A scoping review was considered appropriate because the topic spans several overlapping areas, including nephrology, disaster medicine, emergency preparedness, public health, nursing, and health services research, and because the available literature appears to be heterogeneous in both design and focus. In contrast to a systematic review, which is generally used to answer a narrowly defined effectiveness question, a scoping review is particularly useful when the objective is to identify key concepts, summarize broad patterns in the literature, clarify how a field has been studied, and highlight gaps for future research [34].

The purpose of the present review was therefore not to determine the effectiveness of a specific intervention, but rather to examine how patient-centered care has been described, addressed, or implied in the context of dialysis care during DPHEs. The review aimed to capture both directly disaster-focused studies and studies that contributed conceptually relevant evidence on patient-centered dialysis care in disrupted or emergency-related conditions. In this way, the review sought to provide a broad mapping of the field and to identify the domains through which patient-centered care may be understood in dialysis-dependent populations during crises.

### Information sources

The literature search was conducted in three electronic databases: Web of Science, Scopus, and PubMed, during April 1 to April 10, 2026. These databases were selected because together they provide broad coverage of biomedical, nursing, public health, and interdisciplinary health services research. Their combined use was intended to maximize retrieval of studies addressing dialysis care, disaster preparedness, emergency response, and patient-centered care from clinical, nursing, organizational, and systems perspectives.

### Search strategy

A structured search strategy was developed to identify studies in English, combining concepts related to disasters or emergencies, patient-centered care, vulnerability, and dialysis, published between 2014 and 2026. Because terminology in this field is variable and because the review question sits at the intersection of several disciplines, the search was intentionally focused enough to capture literature on both direct disaster response and conceptually related patient-centered dialysis care.

The Web of Science search string was:

- ("Disaster") AND ("Emergency") AND ("Patient-centered") AND ("Care") AND ("Vulnerable") AND ("Dialysis") AND (“Alternative care facility")

The Scopus search string was:

- ("Disaster" AND "Emergency") AND "Patient-centered" AND "Care" AND "Dialysis"

The PubMed search string was:

- ("Disaster" OR "Emergency") AND "Patient-centered" AND "Care" AND "Dialysis"

The search strategy reflected the conceptual focus of the review rather than a narrow intervention-based framework. The inclusion of terms such as disaster, emergency, patient-centered care, vulnerable, dialysis, and alternative care facility was intended to identify literature relevant to disruptions in dialysis care and to the broader question of how patient-centered care is maintained under crisis conditions. Because the literature in this area is dispersed and does not always explicitly use the term patient-centered care, the search strategy used diverse keywords and keyword combinations to maximize retrieval of potentially relevant records across databases. Differences between search strings reflected database-specific indexing, search functions, and syntax requirements rather than different review objectives. The search results were subsequently interpreted with a degree of conceptual flexibility during screening. In other words, the search strategy was intentionally focused to prioritize literature most directly relevant to dialysis-dependent patients in disaster and public health emergency contexts, rather than to maximize retrieval of all broader kidney care or emergency care literature.

### Eligibility criteria

Studies were eligible for inclusion if they met the following criteria:

1. They addressed patients receiving dialysis or requiring dialysis-related kidney care, including hemodialysis, peritoneal dialysis, or broader kidney care contexts clearly relevant to dialysis-dependent populations,
2. They focused on disasters, emergencies, pandemics, outbreaks, or major disruptions to care delivery, or contributed conceptually relevant evidence on patient-centered dialysis care that could reasonably inform emergency or disrupted-care contexts,
3. They addressed one or more domains relevant to patient-centered care, such as preparedness, continuity of care, treatment access, communication, resilience, patient experience, psychosocial support, family involvement, telehealth, or care adaptation under constrained conditions, and.
4. They were published in English.

The review was intentionally limited to studies published between 2014 and 2026 to maintain conceptual relevance and manageability in a rapidly expanding literature. Studies not explicitly focused on disasters were included only when they addressed patient-centered dialysis care domains with clear conceptual relevance to disrupted-care or emergency contexts.

Studies were excluded if they focused exclusively on routine dialysis care without any relevance to emergency conditions or to patient-centered domains that could be meaningfully transferred to disaster or public health emergency settings. Records were also excluded if their focus was too general, unrelated to dialysis populations, or not sufficiently connected to the review objective.

Given the exploratory purpose of the review, a broad range of study designs was considered eligible. These included reviews, scoping reviews, qualitative studies, observational studies, case studies, pilot studies, program reports, conceptual papers, clinical trials, and study protocols. This broad inclusion strategy was consistent with scoping review methodology and allowed the review to capture both empirical evidence and conceptual contributions.

### Study selection

Records identified through the database searches were screened in several stages. First, titles were reviewed to identify potentially relevant studies. Where title alone did not provide sufficient information to determine eligibility, abstracts were examined. Full texts were consulted where additional clarification was needed regarding relevance to dialysis care, disaster or emergency contexts, or patient-centered domains. Both authors independently reviewed titles, abstracts, assessed full-text eligibility, and participated in the final selection of included studies. To enhance consistency, a predefined set of eligibility criteria and a stable conceptual framework were applied throughout the selection process. Any uncertainties arising during screening were resolved through discussion and consensus. Borderline records were discussed and a final inclusion decision was made after consensus.

Duplicates identified across databases were removed prior to final inclusion. Screening decisions were guided by the review objective of mapping literature relevant to patient-centered dialysis care in DPHEs, rather than by strict intervention-based criteria. As a result, some studies were retained not because they explicitly addressed disasters, but because they contributed conceptually important evidence on patient-centered care dimensions highly relevant to dialysis patients during emergency conditions, such as telehealth, psychosocial support, advance care planning, and patient-centered care models.

The final review included 43 studies judged to be relevant to dialysis care in DPHEs or to patient-centered dialysis care domains with clear applicability to such contexts. The PRISMA-ScR Checklist is provided as supplementary material. To address the conceptual breadth of the topic, screening was conducted with attention to both explicit disaster-focused studies and studies contributing patient-centered dialysis care domains with clear relevance to emergency or disrupted-care settings.

### Data charting

A data-charting approach was used to extract and organize key information from each included study. For each record, the following information was charted:

- Title of the study,
- Database source,
- Provisional study type or design,
- Disaster, emergency, or care disruption context,
- Dialysis modality or kidney care population, and.
- The study’s relevance to patient-centered care.

Data charting was conducted by the authors using the same extraction structure across all included studies. A summary of the search strategy and data charting framework is presented in Table 1. To support consistency, charting categories were kept stable throughout the review process, and previously charted records were revisited when needed to ensure alignment with later extraction decisions.

**Table 1:** Summary of search strategy and data charting framework

Component	Description
Databases searched	Web of Science, Scopus, PubMed
Search date(s)	April 1 to April 10, 2026
Core search concepts	Disaster/emergency; patient-centered care; vulnerability; dialysis; alternative care facility
Search approach	Focused, concept-driven search
Inclusion criteria	Dialysis-related populations; emergency/disaster or conceptually relevant disrupted-care context; patient-centered domains; English language
Exclusion criteria	Routine dialysis care without emergency relevance; overly general or unrelated studies
Data charting items	Title; database source; study type/design; context; dialysis modality/population; relevance to patient-centered care

The authors also reviewed the extraction structure jointly before full charting to ensure consistent interpretation of data items. This charting process was intended to support a structured overview of the literature rather than a formal risk-of-bias or quality appraisal. Because the purpose of the review was mapping rather than effectiveness evaluation, extracted data were used primarily to identify common themes, conceptual emphases, and areas of concentration or absence in the literature.

### Data synthesis

The included studies were synthesized descriptively and organized thematically [35]. Thematic organization was chosen because of the heterogeneity of study designs and the conceptual breadth of the review question. Rather than pooling results quantitatively, the review focused on identifying recurring patterns in how the literature addressed patient-centered care in the context of dialysis and emergency conditions.

Through this descriptive synthesis, studies were grouped into broad thematic categories (see the Results section). This thematic approach enabled the review to examine both explicit and implicit representations of patient-centered care. In many cases, studies did not use the term patient-centered care directly, but still addressed domains central to the concept, such as communication, continuity, access, caregiver involvement, psychosocial burden, and adaptation to patients’ circumstances. Organizing the findings thematically therefore allowed these contributions to be recognized within a broader conceptual understanding of patient-centered care in dialysis-dependent populations during DPHEs.

### Methodological considerations

No formal methodological quality assessment was undertaken, as the primary purpose of the review was to map the literature rather than to determine the strength of evidence for specific interventions. This is consistent with the aims of scoping review methodology, especially in emerging or conceptually diffuse fields where the priority is to identify how the topic has been studied and where important gaps remain. At the same time, the review acknowledges that the search strategy was highly specific and that included studies varied in the degree to which they explicitly addressed patient-centered care or emergency conditions. To address this heterogenicity, the review drew together evidence from multiple databases and applied a broad conceptual lens during screening, allowing both directly disaster-focused studies and conceptually relevant patient-centered dialysis studies to be considered. The use of explicit eligibility criteria, structured charting, and repeated consistency checks during screening and synthesis also helped to reduce the risk of selection bias. The included literature ranged from randomized trials and qualitative studies to commentaries, program reports, and conceptual papers; accordingly, the findings should be interpreted as a conceptual and thematic mapping of the field rather than as a definitive synthesis of effectiveness, best practice, or equivalent forms of evidence.

## Results

**Fig. 1:**
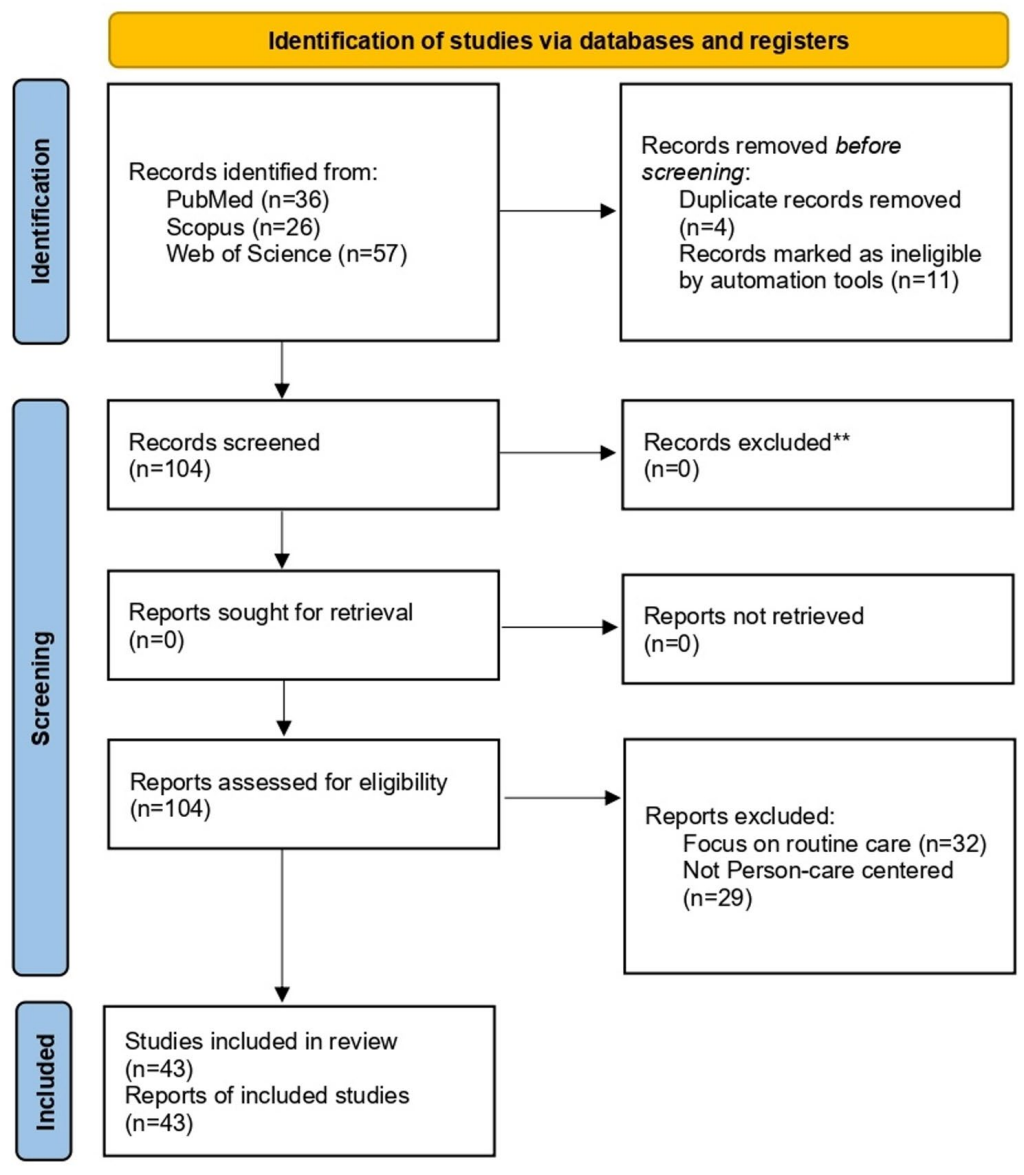
The PRISMA flowchart showing the process of literature selection

The study selection process is summarized in Fig. 1. Database searching identified records 119 from Web of Science (*n* = 57), Scopus (*n* = 26), and PubMed (*n* = 36), which were screened after removal of duplicates (*n* = 4),. Following title and abstract screening, full texts were assessed for eligibility. After exclusion of records that did not meet the inclusion criteria, a total of **43 studies** were included in the scoping review.

### Overview of included studies

The included literature comprised reviews, scoping reviews, qualitative studies, observational studies, case studies, pilot studies, program reports, randomized trials, protocols, and conceptual papers. Most directly disaster-focused papers were identified through Web of Science, while Scopus and PubMed contributed additional literature on COVID-19, telehealth, supportive care, patient experience, and patient-centered models relevant to dialysis populations.

Across the included studies, six broad themes emerged: (a) preparedness for dialysis patients and providers; (b) continuity of care and treatment access during service disruption; (c) infrastructure, logistics, and resilience; (d) patient awareness, communication, and lived experience; (e) public health emergency adaptations, including COVID-19; and (f) conceptual and system-level frameworks for kidney care disaster risk reduction. Collectively, these themes show that patient-centered dialysis care in emergencies is shaped not only by clinical treatment needs, but also by the organization and adaptability of the wider health system. Supplementary Table 1 summarizes diverse items for each included study.

### Preparedness for patients and providers

Preparedness was one of the most prominent themes across the included literature. Several studies focused on disaster planning for dialysis patients, dialysis services, and kidney care providers, including preparedness education, emergency response planning, and professional guidance for both patients and staff [5, 14, 24–26, 28, 36, 37]. These studies indicate that preparedness is a central component of dialysis care in emergencies because treatment continuity depends heavily on advance planning [11, 38]. Preparedness was described at multiple levels, including patient awareness, provider readiness, and organizational planning. Some studies emphasized individual preparedness measures, such as patient knowledge of emergency procedures and treatment arrangements, while others focused on service-level readiness, including nursing roles, provider coordination, and emergency support systems. Taken together, these literatures suggest that preparedness is not only a technical requirement, but also a patient-centered issue, since poor preparedness may leave patients uncertain, unsupported, and at greater risk during disruption.

### Continuity of care and treatment access

Continuity of care and treatment access was another dominant theme in the literature. Several studies examined disrupted dialysis schedules, emergency dialysis starts, facility capacity loss, and treatment delays following disasters such as Hurricane Sandy and Hurricane Maria [6, 10, 12, 15, 39–42]. These studies consistently show that even short interruptions in dialysis care can have serious consequences for patient safety and outcomes [17]. The literature also highlights that continuity of care is shaped by both clinical and logistical factors. Access to functioning facilities, transport, staffing, and coordination all influence whether patients can receive timely treatment. In this way, continuity of care emerges as one of the clearest areas in which operational preparedness and patient-centered care intersect, since maintaining treatment access is fundamental to both survival and patient security during emergencies.

### Infrastructure, logistics, and resilience

Several studies approached dialysis care during disasters from an operational or systems perspective, focusing on logistics, infrastructure, service capacity, and resilience planning [13, 29, 41, 43, 44]. These studies addressed issues such as treatment scheduling, supply chain disruption, emergency support models, and the management of service failure or reduced unit capacity. Although these studies were not always framed in explicitly patient-centered terms, they are highly relevant because dialysis care depends on complex infrastructure. Stable electricity, water supply, transport, consumables, and coordinated staffing are all prerequisites for safe treatment. The literature therefore suggests that resilience in dialysis services is a foundational condition for protecting patients during disasters, even when the focus of the study is organizational rather than experiential [4, 19, 23].

### Patient awareness, communication, and lived experience

A smaller but important body of literature addressed patient awareness, communication, and lived experience more directly. These studies included work on preparedness awareness among hemodialysis patients, qualitative accounts of patient experiences during COVID-19, and broader perspectives on the experiences of patients and providers in disrupted care settings [45, 46]. Other studies contributed insight into advance care planning, peer support, social isolation, and patient-centered care models in dialysis populations [1, 18, 20, 21, 47].

Compared with the preparedness and logistics literature, this group of studies focused more clearly on the human experience of dialysis care. Recurring issues included understanding emergency arrangements, coping with uncertainty, quality of life, psychosocial burden, and the role of support from family, peers, and providers. These studies are particularly relevant to the review question because they show how patient-centered care may be expressed through communication, support, and responsiveness to lived experience during disruptions.

### Public health emergencies and COVID-19

Public health emergency literature, particularly studies from the COVID-19 period, added an important dimension to the review. These studies examined hygiene behaviors, protective attitudes, access to dialysis, telehealth, and the impact of pandemic conditions on quality of life and patient-provider interactions [22, 30–32]. Together, they show that public health emergencies create distinct forms of disruption compared with sudden-onset natural hazard-induced disasters.

Rather than focusing mainly on infrastructure failure, COVID-19-related studies highlighted prolonged uncertainty, infection risk, social isolation, behavioral adaptation, and changes in care delivery. This suggests that patient-centered care in public health emergencies includes not only continuity of treatment, but also support for communication, emotional well-being, infection-related concerns, and new models of remote or home-based care [8, 19].

### Frameworks for kidney care disaster risk reduction

Several studies offered broader conceptual and framework-based perspectives on kidney care in disasters, highlighting the need to move beyond short-term emergency response toward longer-term preparedness, resilience, and risk reduction [7, 48]. Rather than treating disasters as isolated events, these papers frame them as recurring threats that should be anticipated through system-level planning. This perspective is especially relevant for dialysis care because treatment depends on stable infrastructure, supply chains, trained staff, transport, and communication. Framework-based work therefore emphasizes that the vulnerability of dialysis patients is not only clinical, but also structural.

Preparedness in kidney care must include contingency planning, service coordination, patient education, and infrastructure resilience, rather than relying only on reactive measures once disruption occurs [7]. Sandal and Jha [48] extend this discussion by linking kidney care planning to climate change adaptation and mitigation, arguing that environmental instability should be considered part of long-term kidney care planning. Together, these studies suggest that disaster preparedness in nephrology should be embedded within routine care systems and designed to protect both treatment continuity and the broader patient experience during future emergencies.

## Discussion

This scoping review suggests that patients requiring dialysis are consistently recognized as one of the most vulnerable groups during DPHEs. The literature clearly shows that dialysis care is highly sensitive to disruption because it depends on recurrent access to specialized treatment, transport, staffing, power, water, supplies, and coordinated communication [5, 11, 13, 38]. Accordingly, preparedness and continuity of care emerged as the dominant themes across the included studies. More specifically, the review suggests that patient-centered care in dialysis emergencies is represented in the literature primarily through preparedness, continuity, communication, and service adaptability, rather than through explicitly defined patient-centered frameworks. Viewed from a person-centered health systems perspective, these findings indicate that the ability to deliver respectful, coordinated, and responsive dialysis care during emergencies depends not only on clinical expertise, but also on the design and functioning of the systems within which care is delivered.

A central finding is that the literature addresses many patient-centered domains but often does so indirectly. Most studies did not explicitly define patient-centered care in theoretical terms. Instead, they focused on the practical conditions that make patient-centered dialysis care possible, including preparedness, continuity of treatment, care coordination, communication, telehealth, and support for home modalities [18, 25, 30, 32]. In this sense, the literature suggests that patient-centered care for dialysis patients in emergencies is inseparable from system preparedness. The review also indicates that patient-centered care should be understood as an equity issue, since patients with fewer social, financial, transport, or caregiving resources are likely to be disproportionately affected when dialysis services are disrupted.

The findings also show that the disaster literature and the public health emergency literature contribute different but complementary perspectives. Disaster-focused studies, especially those related to hurricanes and service collapse, emphasize treatment interruption, emergency scheduling, transport, mortality risk, and infrastructure resilience [10, 12, 14, 15, 17, 39]. COVID-19-related studies highlight quality of life, infection control, patient behavior, social isolation, dialysis access restrictions, and telehealth [22, 31, 32]. Together, these literatures indicate that patient-centered emergency dialysis care includes not only preserving treatment availability, but also preserving communication, trust, psychosocial support, and individualized adaptation [45, 50]. They also suggest that patient-centered priorities may vary across phases of emergency management, from preparedness and acute response to recovery and restoration of routine care.

These findings also suggest that patient-centered dialysis care should be understood as a health systems and policy issue, rather than only a clinical or operational one. Preparedness planning, service coordination, communication, telehealth, and infrastructure resilience all function as system-level enablers of person-centered care. When these elements are absent or weak, patients may still receive technically necessary treatment, but in ways that are fragmented, inequitable, or poorly aligned with their lived needs. This suggests that sustaining person-centered care in emergencies depends on whether health systems are designed to protect not only treatment continuity, but also equity, communication, dignity, and support for vulnerable populations [3]. This has clear relevance to SDG 3, which emphasizes access to safe and effective care, and to SDG 10, since patients with fewer resources are likely to experience the greatest harm when dialysis services are disrupted [33].

Another important finding is the relative scarcity of explicit patient-centered outcome measures. Even in studies that were highly relevant to patient-centered domains, the emphasis was often on logistics, safety, and preparedness rather than on patient preferences, shared decision-making, caregiver involvement, or perceived dignity. In addition, much of the literature remains descriptive, retrospective, or framework-based, limiting firm conclusions about which patient-centered strategies are most effective under different emergency conditions [16, 49]. This gap suggests that the field is ready for the development of candidate indicators for patient-centered dialysis care in emergencies. The mainly descriptive character of the findings reflects both the scoping review design and the nature of the available literature, which remains dominated by reviews, case reports, observational studies, and conceptual papers rather than comparative intervention research.

### Alternative care facilities and hotel-based models

One future planning consideration arising from the broader challenge of continuity and system adaptability is the possible role of alternative care facilities (ACFs) in supporting dialysis patients during DPHEs. General disaster planning often considers ACFs as surge solutions when hospitals are overwhelmed or infrastructure is disrupted [50]. For dialysis populations, however, the use of ACFs is more complicated than for general low-acuity surge care. In-center hemodialysis requires highly specific infrastructure, including water treatment systems, reliable electricity, machines, consumables, infection prevention capacity, trained dialysis personnel, and emergency backup arrangements [13, 38].

Peritoneal dialysis may be somewhat more adaptable in decentralized environments, but it still depends on secure storage, sterile supplies, adequate hygiene, patient training, and communication with clinical teams [36, 44]. Therefore, a hotel or other repurposed facility is unlikely to serve as a fully functional dialysis unit unless it has been specifically modified and integrated with nephrology expertise and technical support [51, 52].

From a patient-centered perspective, the relevance of ACFs lies not simply in bed expansion, but in whether they preserve continuity, safety, dignity, practical access, and communication. A hotel-based facility could potentially support displaced dialysis patients through temporary lodging near functioning dialysis centers, coordination of transport, monitoring of medically stable patients, or support for home dialysis patients whose housing has been disrupted. In contrast, using such a facility as a stand-alone site for direct dialysis delivery would require substantial infrastructure and oversight, and may not be feasible in most settings [51].

The reviewed literature did not provide strong direct evidence on hotel-based ACFs specifically for dialysis. Nevertheless, the themes of service resilience, supply vulnerability, decentralized support, and continuity planning suggest that ACFs may have a role within a tiered continuity model. In such a model, the least infrastructure-intensive functions, such as lodging, coordination, observation, telehealth support, and low-acuity care, could potentially be shifted to alternative sites, while dialysis delivery itself would remain linked to dedicated dialysis-capable environments whenever possible. This is likely the safest and most patient-centered interpretation at present.

### Implications for research and practice

The findings support at least three next steps. First, future research should explicitly define what patient-centered care means for dialysis patients in emergencies and how it can be operationalized at patient, service, and system levels. Second, the field would benefit from developing candidate indicators focused on communication, preparedness, continuity planning, family and caregiver involvement, psychosocial support, equity, and respect for patient preferences. Third, health systems should consider how emergency plans for dialysis populations can incorporate both infrastructure resilience and person-centered supports, including telehealth, decentralized coordination, and selective use of alternative care facilities. More broadly, emergency preparedness for dialysis populations should be evaluated not only by whether treatment was maintained, but also by whether systems supported understandable communication, equitable access, continuity planning, and the relational dimensions of care.

### Limitations

This review has several limitations. First, the search strategies were narrow and relied on specific combinations of terms such as disaster, emergency, patient-centered, vulnerable, dialysis, and alternative care facility. This reflected an intentional effort to maintain conceptual focus on dialysis-dependent patients in disaster and public health emergency contexts, but it may have reduced the sensitivity of the search. Relevant studies may therefore have been missed if they addressed service disruption, preparedness, or continuity without using those exact terms. To reduce this risk, searches were conducted across three major databases with complementary coverage, and screening was performed with conceptual flexibility so that studies were not excluded solely because they did not explicitly use the term patient-centered care.

Second, the search strings differed across databases, which may have contributed to heterogeneity in the included records. However, this was partly mitigated by applying consistent eligibility criteria across all records during screening and by using the same review objective to guide study selection across databases.

Third, some included studies were retained because of their conceptual relevance to patient-centered dialysis care rather than their explicit focus on disasters. This broadens the scope of the review and introduces variation in direct relevance, but it was a deliberate strategy consistent with scoping review methodology and the exploratory aim of identifying patient-centered care domains applicable to emergency conditions.

Fourth, the evidence on ACFs for dialysis appears limited and indirect, so conclusions in that area remain preliminary. To address this, the discussion interprets ACFs cautiously and restricts conclusions to supportive or selective roles rather than assuming full dialysis equivalence.

Finally, this scoping review mapped the literature but did not formally assess study quality or intervention effectiveness. This was consistent with the aim of the review, which was to examine the extent and nature of the literature rather than to determine the superiority of particular strategies.

## Conclusion

Patients requiring dialysis are among the most vulnerable populations during disasters and public health emergencies because their survival depends on uninterrupted access to complex and resource-intensive care. The literature identified in this review consistently emphasizes preparedness, continuity of care, infrastructure resilience, and adaptation across both natural hazard-induced disasters and public health emergencies. Although the term patient-centered care is used only inconsistently, many included studies address its practical dimensions indirectly, particularly communication, planning, access, continuity, psychosocial support, and care coordination.

Overall, the current literature provides an important foundation, but it leaves a significant gap in the explicit definition and operationalization of patient-centered dialysis care in emergency settings. Viewed through a person-centered health systems lens, the findings suggest that dialysis preparedness should be assessed not only by whether treatment delivery is preserved, but also by whether health systems sustain communication, equity, dignity, support, and continuity for those most at risk of harm. Future research should build on this foundation to identify measurable indicators and practical frameworks that preserve both treatment continuity and the human dimensions of care (Appendix A). Alternative care facilities, including hotel-based models, may have a limited but potentially useful role in supporting dialysis continuity, especially for lodging, coordination, and low-acuity support, but they are not straightforward substitutes for dialysis-capable infrastructure.

## Supplementary Information

Below is the link to the electronic supplementary material.

Supplementary Material 1: Appendix: PRISMA Checklist; Supplementary Table 1

## Data Availability

All data generated or analyzed during this study are included in this published article and its supplementary information files. The search strategies, study selection details, and extracted thematic categories are available from the corresponding author on reasonable request.

## Abbreviations

ACF: Alternative Care Facility
COVID-19: Coronavirus disease 2019
DPHEs: Disasters and Public Health Emergencies
PCC: Patient-Centered Care
SDG: Sustainable Development Goal
