# Supplementary material for: Patient-centered care for patients requiring dialysis during disasters and public health emergencies: a scoping review
Source: Int J Emerg Med. 2026 May 29;19:149. doi: 10.1186/s12245-026-01253-7 (PMC13224643; doi:10.1186/s12245-026-01253-7)
Supplement: Supplementary file 1 — Supplementary Material 1: Appendix: PRISMA Checklist; Supplementary Table 1 [file 12245_2026_1253_MOESM1_ESM.docx]

**Supplementary Table 1:** The summary of included papers. Several entries are **not directly disaster-focused**, but they still have value for this review because they inform patient-centered domains such as care coordination, peer support, advance care planning, telehealth, or measurement. Furthermore, there is a heterogenicity in obtained studiesa, limiting their scientific weight, although still useful in a scoping review.

| No. | Title | Author / Journal | Study type and publication year | Key points | Comments and scientific value |
| --- | --- | --- | --- | --- | --- |
| 1 | Lessons learnt and future directions in managing dialysis access during the COVID-19 pandemic: Patient and provider experience in the United States | Agarwal et al. / *Journal of Vascular Access* | Experience report / review, 2023 | Examines dialysis access challenges during COVID-19 from patient and provider perspectives; highlights service adaptation and access continuity. | Valuable for linking patient and provider experience to emergency service redesign; relevant to patient-centered access but likely limited by descriptive design. |
| 2 | Improving primary care delivery for patients receiving maintenance hemodialysis | Beers et al. / *American Journal of Kidney Diseases* | Health services / care model paper, 2021 | Discusses ways to strengthen primary care integration for hemodialysis patients. | Scientifically useful for patient-centered coordination and continuity; indirectly relevant to disasters by showing baseline system needs. |
| 3 | Hygiene behaviours and protective attitudes in haemodialysis patients during COVID-19: impact on quality of life | Bicakci et al. / *BMC Nephrology* | Observational study, 2026 | Explores infection-related behaviors and their association with quality of life during COVID-19. | Useful for patient experience in public health emergencies; adds patient-centered outcome content, though disaster focus is pandemic-specific. |
| 4 | Hurricanes and mortality among patients receiving dialysis | Blum et al. / *Journal of the American Society of Nephrology* | Observational epidemiological study, 2022 | Links hurricane exposure to mortality risk among dialysis patients. | Strong scientific value for quantifying disaster-related risk; important for preparedness policy and vulnerability framing. |
| 5 | Disaster management in a nephrology service: lessons learned from Hurricane Maria | Bonilla-Félix & Suárez-Rivera / *Blood Purification* | Case report / service report, 2019 | Describes nephrology service response and lessons from Hurricane Maria. | High practical relevance; useful for real-world preparedness and continuity lessons, though limited generalizability. |
| 6 | Dialysis in disaster: Using continuous renal replacement therapy for end-stage renal disease patients, a pilot proof of concept study | Boparai et al. / *American Journal of Emergency Medicine* | Pilot proof-of-concept study, 2022 | Tests CRRT as a possible substitute option for ESRD patients during disaster conditions. | Innovative and practically important; limited by pilot nature, but highly relevant to surge and contingency planning. |
| 7 | Experiences of individuals undergoing maintenance hemodialysis during the COVID-19 pandemic in China | Dai et al. / *BMC Nephrology* | Qualitative study, 2025 | Explores lived experience of maintenance hemodialysis patients during COVID-19. | Strong patient-centered value; useful for communication, psychosocial burden, and coping. |
| 8 | The impact of disasters on populations with health and health care disparities | Davis et al. / *Disaster Medicine and Public Health Preparedness* | Review / conceptual paper, 2010 | Addresses how disasters disproportionately affect populations with health disparities. | Important contextual paper for vulnerability framing; not dialysis-specific, but valuable conceptual support. |
| 9 | Progress in emergency preparedness for dialysis care 10 years after Hurricane Katrina | Dent et al. / *American Journal of Kidney Diseases* | Commentary / review, 2015 | Reflects on progress and remaining gaps in dialysis disaster preparedness after Katrina. | Useful historical benchmark; important for preparedness systems perspective. |
| 10 | Hurricane Helene's Impact on Peritoneal Dialysis Supply Chain: A Case Study in Healthcare System Vulnerability | El Shamy & Shah / *Kidney 360* | Case study, 2025 | Highlights how a hurricane disrupted peritoneal dialysis supply chains. | Highly relevant to resilience and home dialysis preparedness; strong practical systems insight. |
| 11 | Rethinking Potentially Preventable Emergency Department Use Among People Receiving Dialysis | Elliott et al. / *Journal of the American Society of Nephrology* | Population-based study / abstract, 2022 | Examines potentially preventable ED use among dialysis patients. | Useful for understanding care coordination and avoidable acute care use; relevance to emergencies is indirect. |
| 12 | Assessing the disruption impact on healthcare delivery | Fatani et al. / *Journal of Primary Care & Community Health* | Health systems study, 2024 | Evaluates how disruptions affect healthcare delivery. | General systems relevance; supportive background for disruption science, but not dialysis-specific. |
| 13 | Barriers and facilitators to provide continuity of care to dischargeable patients in disasters | Feizolahzadeh et al. / *Injury* | Qualitative study, 2019 | Identifies barriers and facilitators to continuity of care during disasters. | Strong conceptual relevance for continuity and systems issues; not dialysis-specific but highly transferable. |
| 14 | Toward patient-centered innovation: a conceptual framework for patient-reported outcome measures for transformative kidney replacement devices | Flythe et al. / *CJASN* | Conceptual framework paper, 2020 | Proposes patient-reported outcome domains for kidney replacement technologies. | High scientific value for operationalizing patient-centered measures; especially relevant to indicator development. |
| 15 | Effect of peer mentorship on hospitalizations among patients receiving maintenance hemodialysis | Golestaneh et al. / *JASN* | Pragmatic randomized controlled trial, 2024 | Tests peer mentorship and its effect on hospitalization. | Strong evidence for relational and supportive interventions; indirect disaster relevance but useful for patient-centered care design. |
| 16 | Evaluating a patient-centered medical home for patients receiving dialysis for kidney disease | Hynes et al. / PCORI | Evaluation / research report, 2019 | Evaluates a patient-centered medical home model for dialysis patients. | Important for care coordination and patient-centered systems; strong relevance despite non-disaster context. |
| 17 | Dialysis care and death following Hurricane Sandy | Kelman et al. / *American Journal of Kidney Diseases* | Observational study, 2015 | Investigates association between disrupted dialysis care and mortality after Hurricane Sandy. | High scientific importance for showing real patient consequences of disruption. |
| 18 | The impact of disasters and major incidents on vulnerable groups: Risk and medical assessment of Swedish patients with advanced care at home | Khorram-Manesh et al. / *Home Health Care Management & Practice* | Observational / vulnerability study, 2017 | Examines disaster impact on medically vulnerable groups receiving advanced home care. | Not dialysis-specific, but useful for vulnerability and home-based care perspectives. |
| 19 | Disaster preparedness for patients with kidney disease | Kopp et al. / *Nature Reviews Nephrology* | Commentary / review, 2023 | Summarizes preparedness advice for kidney patients. | High relevance for patient education and preparedness planning; brief but influential. |
| 20 | Renal failure patients in disasters | Lempert & Kopp / *Disaster Medicine and Public Health Preparedness* | Review, 2019 | Reviews issues affecting renal failure patients in disasters. | Foundational background review; directly relevant to dialysis vulnerability and disaster planning. |
| 21 | Operationalizing telehealth for home dialysis patients in the United States | Lew & Sikka / *American Journal of Kidney Diseases* | Practice / implementation paper, 2019 | Discusses telehealth use for home dialysis care. | Scientifically valuable for remote continuity and care flexibility; highly relevant for emergencies and ACF alternatives. |
| 22 | Disaster preparedness and response for hemodialysis nursing: Recent advances and future directions | Liao et al. / *International Journal of Mental Health Nursing* | Conference/short review item, 2024 | Focuses on preparedness and response roles in hemodialysis nursing. | Relevant to workforce and service preparedness; likely limited detail depending on publication format. |
| 23 | Early dialysis and adverse outcomes after Hurricane Sandy | Lurie et al. / *American Journal of Kidney Diseases* | Observational study, 2015 | Examines relation between early dialysis patterns and adverse outcomes after Sandy. | Strong disaster outcome evidence; useful for timing, care disruption, and mortality risk. |
| 24 | Comparative effectiveness of an individualized model of hemodialysis vs conventional hemodialysis: the TwoPlus trial | Murea et al. / *Trials* | Study protocol, 2024 | Describes protocol for testing individualized hemodialysis. | Methodologically useful for individualized care models; indirect disaster relevance but supports patient-centered framing. |
| 25 | Emergency and natural disaster preparedness tips for kidney patients | National Kidney Foundation / NKF | Practice guidance, 2021 | Provides patient-facing preparedness advice for kidney patients. | Important practical resource; limited scientific rigor but useful applied value. |
| 26 | Factors related to preparedness for emergency hemodialysis in the event of a natural disaster | Nihonyanagi et al. / *Medicine* | Observational study, 2022 | Identifies factors associated with preparedness for emergency hemodialysis. | Directly relevant to preparedness determinants; useful for targeted intervention planning. |
| 27 | Integrated treatment scheduling and logistics planning for a hemodialysis center after a disaster with robust travel times | Ozmemis et al. / *Transportation Research Part E* | Operations research / modeling study, 2025 | Develops logistics and scheduling model for post-disaster hemodialysis operations. | Strong systems and logistics value; less patient-experiential, but crucial for continuity planning. |
| 28 | Rationale and design of a patient-centered medical home intervention for patients with ESRD on hemodialysis | Porter et al. / *Contemporary Clinical Trials* | Trial design / protocol, 2015 | Outlines design of a patient-centered medical home intervention. | Scientifically useful for conceptualizing coordinated, patient-centered dialysis care. |
| 29 | The mechanisms that shape the care trajectory leading to an emergency dialysis start | Raffray et al. / *JASN* | Abstract / qualitative-perspective study, 2022 | Explores perspectives shaping emergency dialysis initiation. | Relevant to patient pathway understanding and delayed care; limited by abstract format. |
| 30 | Avoiding a dialysis unit disaster: Managing a prolonged and near-complete loss of capacity at a major dialysis hub | Randall et al. / *Hemodialysis International* | Case report / service management study, 2022 | Describes management of severe dialysis hub capacity loss. | Highly relevant applied evidence for contingency operations and resilience. |
| 31 | Enhancing disaster preparedness in peritoneal dialysis care | Sahutoglu & Kazancioglu / *Kidney International Reports* | Commentary / review, 2024 | Focuses on preparedness in peritoneal dialysis. | Useful for home-based modality preparedness and decentralized care. |
| 32 | Time to integrate climate science into kidney care planning: a climate change mitigation and adaptation framework | Sandal & Jha / *Current Opinion in Nephrology and Hypertension* | Conceptual / framework paper, 2025 | Connects climate adaptation with kidney care planning. | Important for forward-looking resilience and systems sustainability. |
| 33 | A roadmap for disaster risk reduction and management in kidney care: a scoping review and content analysis | Sandal et al. / *JASN* | Scoping review / content analysis, 2025 | Proposes a structured roadmap for kidney disaster risk reduction. | High scientific and conceptual value; likely one of the strongest framework papers in the field. |
| 34 | Disasters and kidney care: Pitfalls and solutions | Sever et al. / *Nature Reviews Nephrology* | Narrative review, 2023 | Reviews major problems and solutions in kidney care during disasters. | High-impact synthesis; strong reference for broad field overview. |
| 35 | Disaster preparedness for people with kidney disease and kidney healthcare providers | Sever et al. / *Current Opinion in Nephrology and Hypertension* | Review, 2024 | Summarizes preparedness considerations for patients and providers. | Strong applied relevance; useful for practice and systems planning. |
| 36 | Dialysis in crisis: Delivering renal replacement therapy in disasters, conflicts, and resource-constrained settings | Sharma et al. / *JPMA* | Review / commentary, 2025 | Discusses dialysis delivery in crises and low-resource settings. | Useful for global and austere-environment perspective; likely concise rather than comprehensive. |
| 37 | Natural disasters in the Americas, dialysis patients, and implications for emergency planning: a systematic review | Smith et al. / *Preventing Chronic Disease* | Systematic review, 2020 | Synthesizes evidence on natural disasters and dialysis patients in the Americas. | High scientific value as a formal review; strong for emergency planning implications. |
| 38 | Effectiveness of an advance care planning intervention in adults receiving dialysis and their families | Song et al. / *JAMA Network Open* | Cluster randomized clinical trial, 2024 | Tests advance care planning in dialysis patients and families. | Strong patient-centered intervention evidence; indirectly relevant to emergencies through planning and caregiver engagement. |
| 39 | Utilization and costs of health care in a kidney supportive care program | Sowa et al. / *Journal of Palliative Care* | Observational health services study, 2020 | Examines use and costs in a kidney supportive care program. | Useful for supportive care and resource implications; indirect emergency relevance. |
| 40 | Building resilience in hemodialysis care: a program report on the British Columbia Hemodialysis Emergency Support Team | Thomas / *Canadian Journal of Kidney Health and Disease* | Program report, 2025 | Describes emergency support team model for hemodialysis resilience. | Strong practical contribution for organized system preparedness and resilience. |
| 41 | Association rules of social alienation in maintenance hemodialysis patients | Wanning et al. / *International Urology and Nephrology* | Observational / analytic study, 2025 | Examines social alienation and intervention strategies in maintenance hemodialysis. | Useful for psychosocial burden and patient experience; indirect disaster relevance. |
| 42 | Patient-centered quality measures for dialysis care: KDOQI scientific workshop report | Weiner et al. / *American Journal of Kidney Diseases* | Workshop report / consensus paper, 2024 | Proposes patient-centered quality measures for dialysis care. | Very strong conceptual and measurement value; directly relevant to indicator development. |
| 43 | Role of big data analytics capability in developing integrated hospital supply chains and operational flexibility | Yu et al. / *Technological Forecasting and Social Change* | Organizational / systems study, 2021 | Examines supply chain integration and flexibility using big data analytics. | Not dialysis-specific and only indirectly relevant; useful mainly for broader logistics and operational resilience concepts. |
